# Hispanic/Latinx ethnic differences in the relationships between behavioral inhibition, anxiety, and substance use in youth from the ABCD cohort

**DOI:** 10.3389/fpsyt.2023.1251032

**Published:** 2023-10-06

**Authors:** Kelly A. Correa, Everett L. Delfel, Alexander L. Wallace, William E. Pelham III, Joanna Jacobus

**Affiliations:** ^1^Department of Psychiatry, University of California San Diego, San Diego, CA, United States; ^2^San Diego State University/University of California (SDSU/UC) San Diego Joint Doctoral Program in Clinical Psychology, San Diego, CA, United States

**Keywords:** behavioral inhibition, behavioral activation, anxiety, substance use, Hispanic, Latinx

## Abstract

**Introduction:**

Elevated levels of behavioral inhibition (BI) may connote risk for both anxiety and substance use disorders. BI has consistently been shown to be associated with increased levels of anxiety, while the association between BI and substance use has been mixed. It is possible that the relationship between BI and substance use varies by individual difference factors. Hispanic/Latinx (H/L) youth in particular may have stronger relationships between BI, anxiety, and substance use.

**Methods:**

The present study therefore evaluated (1) the prospective relationships between BI [assessed via self-reported behavioral inhibition system (BIS) scale scores], anxiety, and substance use in youth (*n* = 11,876) across baseline, 1-, and 2-year follow-ups of the Adolescent Brain Cognitive Development (ABCD) Study (ages 9–12) and (2) whether these relationships differed by H/L ethnicity while covarying for average behavioral approach system scores, race, sex, age, highest parental income, highest parental education, and past-year substance use (for analyses involving substance use outcomes).

**Results:**

Baseline levels of BIS scores predicted increased anxiety symptoms at both 1- and 2-year follow-ups and did not differ by H/L ethnicity. Baseline levels of BIS scores also prospectively predicted increased likelihood of substance use at 2-year follow-up, but only for H/L youth and not at 1-year follow-up.

**Discussion:**

High scores on the BIS scale contribute risk to anxiety across ethnicities and may uniquely contribute to risk for substance use in H/L youth.

## Introduction

Anxiety disorders and substance use disorders are highly prevalent and frequently co-occur (1–3). Anxiety disorders are associated with significant quality-of-life impairments, carry an economic burden of billions of dollars in the United States, and are one of the top ten leading causes of disability globally (4–7). When anxiety disorders co-occur with substance use disorders, such co-occurrence is associated with markedly worse outcomes such as increased rates of drug-related problems, unemployment, and poorer treatment outcomes (8–11). The onset of both anxiety and substance use disorders often occurs during childhood and adolescence (3) and understanding the potential shared etiological mechanisms underlying both anxiety and substance use disorders may help improve assessment and treatment of these disorders and their co-occurrence.

Gray’s reinforcement sensitivity theory (RST) (12–14) offers explanations of risk for both disorders and possibly also for their co-occurrence. RST describes two major neurophysiological motivational systems that differ in their responsivity to reward and punishment. The behavioral inhibition system (BIS) is activated when punishment or non-reward conflicts with a goal or reward and results in worry, risk assessment, and increased attention to threat. In contrast, the behavioral approach/activation system (BAS) is activated in response to rewarding stimuli and results in impulsivity, goal pursuit, and increased attention to reward. The biologically based temperamental style of BI is similar to the BIS in that it is characterized by anxious, fearful, and vigilant reactions to novel stimuli (15–17). BI is identifiable as early as infancy and has demonstrated trait-like stability (18). Several studies have shown BI (assessed via behavioral observation in infancy or early childhood or maternal ratings across early childhood) to be stable across early and middle childhood and possibly through late adolescence and early adulthood (15, 19–21). However, the stability and predictive validity of BI varies across individuals. For example, BI (assessed via maternal report) is more stable for girls and those who are initially highest in BI as compared to their peers (20). Further, many of the infants and toddlers that are initially high in behaviorally assessed BI no longer display such sensitivity to novel stimuli as they age (15, 22). Degnan et al. (22) showed that only 15% of toddlers displayed initially high levels of BI and continued to display such high levels of BI at age 5. Identification of children with high and stable levels of BI (assessed via behavioral observation and/or maternal report) is particularly important as they are at increased risk for experiencing symptoms of anxiety and the development of anxiety disorders in adolescence (17, 23–28). In fact, nearly half of children high in BI develop social anxiety disorder in adolescence (25). The BIS may be related to and influenced by BI. While the BIS is assessed by self-report and at later stages of childhood through adulthood, as compared to the behavioral assessment of BI, scores on the BIS/BAS scale may yield improved reliability and generalizability in its assessment of BI (29–31).

The relationship between BI and substance use is much more mixed than that of BI and anxiety. BI may be a protective factor against substance use as the conflicting rewarding (e.g., conformity, relief from distress, etc.) and punishing (e.g., negative health consequences, family conflict, etc.) outcomes associated with substance use may activate the BIS and lead to worry and increased attention to the potential negative consequences of use. However, it is also possible that BI may increase the likelihood of substance use through coping motives for use (32). Individuals with alcohol use disorder as well as individuals with co-occurring anxiety and alcohol dependence evidence greater levels of sensitivity to uncertain threat (33–36), a trait associated with, and predicted by, high levels of BI (37). These high levels of sensitivity to uncertain threat in individuals with alcohol use disorder are also positively associated with self-reported coping motives for use.

To further explore BI’s potential risk for, or protection against, substance use, studies have examined BIS and BAS levels on substance use outcomes. These studies have focused on undergraduate populations and yielded mixed results with some studies showing no association between BIS levels and substance use (38, 39), others showing a positive association between BIS levels with substance use problems (40–42), and still others showing a positive association between BIS levels and substance use but only for high BAS levels (41–43). Given these conflicting results, Morris et al. (40) used a cross-sectional design to examine whether BIS and BAS were indirectly associated with alcohol problems through coping and conformity motives among undergraduate students. Results indicated that those high in BIS levels were more likely to experience alcohol problems due to greater coping and conformity motives for use. Importantly this finding was independent of levels of BAS and high BAS levels only further strengthened these relationships. Taken together, these results highlight BI’s nuanced pathways for high or low risk for substance use and demonstrate the need to investigate potential additional factors contributing to the relationships between BI and substance use.

Ethnicity may be one such important moderator of the relationships between BI, anxiety, and substance use. Hispanic/Latinx (H/L) youth have consistently displayed increased rates of anxiety symptoms, anxiety disorders, and initial rates of substance use when compared to their non-H/L peers (44–51). The greater frequency and intensity for which H/L youth experience threats including increased exposure to crime, community violence, chronic stress, and racial discrimination may heighten levels of BI in H/L youth (52–56). In fact, H/L adults have displayed increased attentional biases to threat as compared to non-H/L adults (57). Cultural values may also further impact BI’s association with anxiety in H/L youth. Schneider and Gudiño (58) showed a positive relationship between BI and anxiety symptoms in H/L adolescents and that this relationship was strongest for those H/L adolescents reporting high levels of Latino cultural values. More specifically, H/L youth may also experience increased anxiety due to heightened social stigma of mental illness in the H/L community and factors related to collectivist cultural values, immigration, and acculturation that, especially in combination, put H/L youth at increased risk when compared to other racial/ethnic groups (46, 50, 59). It is possible that the combination of increased exposure to stressors and traumatic experiences as well as the context of heightened social stigma and collectivist cultural values may dissuade H/L youth from utilizing social support as a form of coping with their anxiety. Since high levels of BI may lead to alcohol problems through coping and conformity motives and H/L youth may experience greater exposure to substance use as they have the highest initial rates of substance use, H/L youth high in BI may be uniquely at risk for substance use. Therefore, H/L ethnicity may moderate the relationship between BI and substance use. However, it is presently unclear whether the strengths of the relationships between BI, anxiety, and substance use differ based on H/L ethnicity. Therefore, the present study will prospectively investigate the relationships between BIS scale scores (used as an approximation of BI), anxiety, and substance use and whether H/L ethnicity moderates such relationships in youth (*n* = 11,876) from the Adolescent Brain Cognitive Development (ABCD) Study at baseline (ages 9–10), 1-year follow-up (ages 10–11), and 2-year follow-up (ages 11–12).

## Materials and methods

### Participants and procedure

Data for the current study was drawn from baseline (ages 9–10), 1-year follow-up (ages 10–11), and 2-year follow-up (ages 11–12) of the ABCD Data Release 4.0. The ABCD Study is a longitudinal study with 11,876 participants, is funded by the National Institutes of Health, and is a probability sample and not a clinical sample (60, 61). Youth and their parent or guardian completed baseline, 1-year follow-up, and 2-year follow-up study visits separately to maintain confidentiality. These study visits lasted 8 h (or were split into two 4-h visits) and consisted of a battery of assessments including questionnaires on demographics, mental health, and substance use (62, 63). Youth and their parent/guardian were financially compensated for their participation. The University of California, San Diego Institutional Review Board approved all aspects of this study for the ABCD Research Study Consortium. Please see Table 1 for participant demographics and characteristics.

**TABLE 1:** Participant demographics and characteristics.

	Not Hispanic *n* = 9,312	Hispanic *n* = 2,411	*p*-value
Age [mean (SD)]	9.93 (0.62)	9.88 (0.63)	0.001
Sex *n* (% Male)	4,857 (52.2%)	1,253 (52%)	0.887
Race *n* (%)			<0.001
Multiracial	1,090 (11.7%)	363 (15.1%)	
White	6,093 (65.4%)	1,342 (55.7%)	
Black or African American	1,764 (18.9%)	87 (3.6%)	
Native American or Alaska Native	38 (0.4%)	23 (1%)	
Native Hawaiian or other Pacific Islander	12 (0.1%)	3 (0.1%)	
Asian	222 (2.4%)	19 (0.8%)	
Other race	57 (0.6%)	455 (18.9%)	
Did not answer	36 (0.4%)	119 (4.9%)	
Highest parental education *n* (%)			<0.001
< High school diploma	221 (2.4%)	358 (14.8%)	
High school diploma/GED	723 (7.8%)	390 (16.2%)	
Some college	2,238 (24%)	808 (33.5%)	
Bachelor’s degree	2,552 (27.4%)	429 (17.8%)	
Postgraduate degree	3,570 (38.3%)	421 (17.5%)	
Did not answer	8 (0.1%)	5 (0.2%)	
Highest parental income *n* (%)			<0.001
<50,000	2,924 (31.4%)	1,334 (55.3%)	
50,000–99,999	3,198 (34.4%)	580 (24.1%)	
> 99,999	2,654 (28.5%)	261 (10.8%)	
Did not answer	534 (5.7%)	236 (9.8%)	
Baseline sum of past-year use days	0.03 (2.02)	0.02 (0.57)	0.744
Year 1 follow-up sum of past-year use days	0.03 (1.55)	0.01 (0.14)	0.456
Year 2 follow-up sum of past-year use days	0.06 (2.43)	0.04 (0.69)	0.721
Baseline BIS scores	9.47 (3.74)	9.71 (3.8)	0.005
Baseline mean BAS scores	6.88 (2.29)	7.24 (2.26)	<0.001
Baseline CBCL DSM-5 anxiety scores	53.39 (6.03)	53.92 (6.49)	<0.001
Year 1 follow-up CBCL DSM-5 anxiety scores	53.48 (6.2)	53.71 (6.31)	0.123
Year 2 follow-up CBCL DSM-5 anxiety scores	53.34 (5.92)	53.4 (5.8)	0.727

### Measures

The parent or legal guardian of the participant was asked about the youth participant’s race and ethnicity during the baseline assessment. The parent/legal guardian answered the following two questions derived from the PhenX toolkit (64) to provide information about the youth participant’s race and ethnicity: “Do you consider the child Hispanic/Latino/Latina?” and “What race do you consider the child to be?.”

Youth participants completed a modified version of the BIS/BAS scale (62, 65, 66). The modified version of the BIS/BAS contained 7 items that corresponded to the BIS scale (baseline α = 0.626, 2-year follow-up α = 0.733) and 13 items that corresponded to the BAS scale (baseline α = 0.831, 2-year follow-up α = 0.861). The BAS scale is comprised of three subscales: reward responsiveness, fun seeking, and drive. A mean BAS scale score was created from the average of these 3 BAS subscales.

The DSM-5 anxiety problems subscale T-score from the Child Behavior Checklist (CBCL) (62, 67) was used to capture anxiety symptomatology as an outcome (baseline α = 0.707, 1-year follow-up α = 0.716, 2-year follow-up α = 0.727). The CBCL was completed by parents/guardians who were asked to report on the youth’s behavior. The CBCL DSM-5 anxiety problems subscale assesses symptoms that are consistent with DSM-5 criteria for Generalized Anxiety Disorder, Separation Anxiety Disorder, Social Anxiety Disorder, and Specific Phobia.

Substance use was assessed via the timeline followback interview (TLFB) (63, 68, 69). Youth participants completed the calendar-based and interviewer-administered TLFB which consisted of a retrospective report of substance use days over the past year. Youth reported on the quantity and frequency of which they used any of the following substances: alcohol, cannabis and cannabinoids, nicotine, cocaine or crack cocaine, cathinones, methamphetamine, 3,4-methylenedioxymethamphetamine (“ecstasy, molly or MDMA”), ketamine, gamma hydroxybutyrate, heroin, hallucinogens, inhalants, prescription stimulants, prescription sedative drugs, prescription opioid pain relievers, and cough or cold medicine containing dextromethorphan. The past-year use sum score was created by summing the total number of days in the past year for which the youth endorsed use of any of the above listed substances. These past-year sum scores were then dichotomized into use in the past year vs. no use in the past year.

### Statistical analysis

Logistic regressions were conducted using the “glm()” function in R to evaluate the impact of baseline BIS scores and the interaction between baseline BIS scores and ethnicity (H/L vs. non-H/L) on (1) past-year substance use at 1-year follow-up (use vs. no use) and (2) past-year substance use at 2-year follow-up (use vs. no use). Linear regressions were conducted using the “lm()” function in R to evaluate the impact of baseline BIS scores and the interaction between baseline BIS scores and ethnicity (H/L vs. non-H/L) on (1) 1-year follow-up CBCL DSM-5 anxiety problems T-scores and (2) 2-year follow-up CBCL DSM-5 anxiety problems T-scores. All analyses were conducted in version 4.2.3 of R. While the majority of youth did not report substance use at baseline (99.57%), 0.50% of H/L youth and 0.42% of non-H/L youth did endorse use at baseline. At 1-year follow-up 0.22% of the sample endorsed any substance use and 0.74% endorsed any substance use at 2-year follow-up. Baseline past-year use days was dichotomized into no past-year substance use and past-year substance use (0 and 1, respectively) and included as a covariate. Past-year substance use at baseline was included in both models in which past-year substance use at follow-up was an outcome. Past year substance use at 1-year follow-up was also included as a dichotomous covariate in the model predicting past-year substance use at 2-year follow-up. To control for their effects on anxiety and substance use, all models included the following covariates: mean baseline BAS scores, race, sex, age, highest parental income, and highest parental education.

## Results

### BIS scores predicting anxiety

After adjusting for baseline BAS scores, race, sex, age, highest parental income, and highest parental education, results indicated a main effect of baseline BIS scores on CBCL DSM-5 anxiety problems scores at the 1-year follow-up (*B* = 0.191, *p* < 0.001), as higher scores on the BIS subscale at baseline were related to greater report of anxiety at 1-year follow-up. No main effect of ethnicity or interaction between ethnicity and baseline BIS scores was found (see Table 2).

**TABLE 2:** Effects of ethnicity and BIS scores on CBCL DSM-5 anxiety problems scores at year 1 follow-up.

	*B*	Standard error	*p*-value
Ethnicity (Hispanic)	0.297	0.412	0.471
Baseline BIS scores	0.191	0.018	<0.001
Baseline mean BAS scores	−0.045	0.028	0.104
Sex (male)	0.875	0.119	0.000
Age	−0.051	0.094	0.587
Highest parental income			
50,000–99,999	−0.716	0.166	<0.001
> 99,999	−1.258	0.191	<0.001
Did not answer	−0.379	0.255	0.138
Race			
White	−0.214	0.183	0.243
Black or African American	−1.664	0.238	<0.001
Native American or Alaska Native	−0.539	0.841	0.521
Native Hawaiian or other Pacific Islander	−1.604	1.654	0.332
Asian	−1.830	0.444	<0.001
Other race	−0.325	0.349	0.351
Did not answer	−1.180	0.565	0.037
Highest parental education			
High school diploma/GED	−0.212	0.341	0.534
Some college	0.404	0.309	0.192
Bachelor’s degree	0.343	0.327	0.293
Postgraduate degree	0.354	0.330	0.283
Did not answer	1.874	1.877	0.318
Ethnicity × baseline BIS scores	−0.050	0.039	0.200

Similarly, after adjusting for the aforementioned covariates, results indicated main effects of baseline BIS scores (*B* = 0.213, *p* < 0.001) and baseline BAS scores (*B* = −0.086, *p* = 0.005) on CBCL DSM-5 anxiety problems scores at the 2-year follow-up, as higher scores on the BIS subscale and lower scores on the BAS subscale at baseline were related to greater report of anxiety at the 2-year follow-up. No main effect of ethnicity or interaction between ethnicity and baseline BIS scores was found (see Table 3).

**TABLE 3:** Effects of ethnicity and BIS scores on CBCL DSM-5 anxiety problems scores at year 2 follow-up.

	*B*	Standard error	*p*-value
Ethnicity	0.394	0.457	0.388
Baseline BIS scores	0.213	0.020	<0.001
Baseline mean BAS scores	−0.086	0.031	0.005
Sex	0.529	0.132	<0.001
Age	0.428	0.105	<0.001
Highest parental income			
50,000–99,999	−0.688	0.183	<0.001
> 99,999	−1.313	0.212	<0.001
Did not answer	−0.011	0.292	0.971
Race			
White	−0.266	0.206	0.197
Black or African American	−1.914	0.275	<0.001
Native American or Alaska Native	−1.687	0.964	0.080
Native Hawaiian or other Pacific Islander	−2.466	1.847	0.182
Asian	−1.538	0.483	0.001
Other race	−0.582	0.390	0.136
Did not answer	−1.627	0.660	0.014
Highest parental education			
High school diploma/GED	−0.511	0.398	0.199
Some college	0.355	0.357	0.319
Bachelor’s degree	0.070	0.374	0.851
Postgraduate degree	0.368	0.378	0.331
Did not answer	0.468	1.783	0.793
Ethnicity × baseline BIS scores	−0.073	0.043	0.091

### BIS scores predicting substance use

After adjusting for baseline BAS scores, race, sex, age, highest parental income, highest parental education, and sum of past-year substance use days at baseline, results indicated that there were no main effects or interactions between ethnicity and baseline BIS scores on past-year substance use at 1-year follow-up (see Table 4).

**TABLE 4:** Effects of ethnicity and BIS scores on substance use at year 1 follow-up.

	Odds ratio	2.5% CI	97.5% CI	Chi^2^ *p*-value
Ethnicity (Hispanic)	1.384	0.089	21.581	0.818
Baseline BIS scores	1.017	0.904	1.144	0.782
Baseline mean BAS scores	1.203	1.002	1.443	0.048
Sex (male)	4.065	1.379	11.978	0.004
Age	2.681	1.327	5.416	0.004
Highest parental income				0.144
50,000–99,999	0.306	0.093	1.001	
> 99,999	0.254	0.046	1.385	
Did not answer	0.755	0.164	3.472	
Race				0.140
White	0.766	0.272	2.160	
Black or African American	0.163	0.030	0.878	
Native American or Alaska Native	0.000	0.000	Inf	
Native Hawaiian or other Pacific Islander	0.000	0.000	Inf	
Asian	0.000	0.000	Inf	
Other race	0.606	0.062	5.885	
Did not answer	3.747	0.631	22.257	
Highest parental education				0.184
High school diploma/GED	1.488	0.143	15.440	
Some college	2.360	0.273	20.372	
Bachelor’s degree	0.434	0.033	5.671	
Postgraduate degree	0.863	0.074	10.006	
Did not answer	0.000	0.000	Inf	
Baseline past-year substance use	7.425	0.923	59.732	0.140
Ethnicity × baseline BIS scores	0.890	0.671	1.181	0.413

**FIGURE 1:**
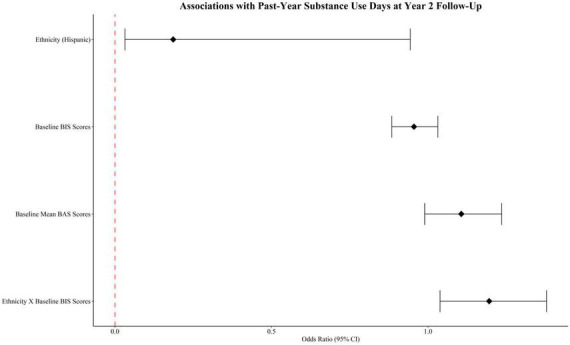
Forest plot of associations with past-year substance use days at year 2 follow-up.

After adjusting for the aforementioned covariates as well as the sum of past-year substance use days at 1-year follow-up, results revealed a main effect of ethnicity [OR = 0.186, 95% CI (0.032, 0.943), *p* = 0.042] such that H/L youth were more likely than non-H/L youth to use substances at the 2-year follow-up. Findings also indicated an interaction between ethnicity and baseline BIS scores [OR = 1.195, 95% CI (1.038, 1.378), *p* = 0.013] on substance use days at year 2 follow-up (see Table 5 and Figure 1). Simple slope analyses were conducted to follow-up the interaction between ethnicity and baseline BIS scores by stratifying by ethnicity. Results showed that BIS scores were associated with increased odds of past-year substance use for H/L youth [*p* = 0.033, 95% CI (1.012, 1.318), OR = 1.155]. However, baseline BIS scores were not associated with odds of past-year substance use for non-H/L youth [*p* = 0.257, 95% CI (0.885, 1.033), OR = 0.956].

**TABLE 5:** Effects of ethnicity and BIS scores on substance use at year 2 follow-up.

	Odds ratio	2.5% CI	97.5% CI	Chi^2^ *p*-value
Ethnicity (Hispanic)	0.186	0.032	0.943	0.042
Baseline BIS scores	0.955	0.884	1.031	0.242
Baseline mean BAS scores	1.106	0.989	1.235	0.076
Sex (male)	0.934	0.575	1.523	0.781
Age	2.012	1.360	3.020	<0.001
Highest parental income				0.337
50,000–99,999	0.625	0.330	1.171	
> 99,999	0.506	0.224	1.105	
Did not answer	0.697	0.198	1.845	
Race				0.175
White	1.673	0.788	4.155	
Black or African American	0.720	0.219	2.305	
Native American or Alaska Native	0.000	0.000	Inf	
Native Hawaiian or other Pacific Islander	0.000	0.000	Inf	
Asian	0.000	0.000	Inf	
Other race	2.940	0.924	9.456	
Did not answer	1.124	0.057	7.168	
Highest parental education				0.482
High school diploma/GED	2.795	0.696	18.676	
Some college	2.166	0.597	13.942	
Bachelor’s degree	2.825	0.731	18.739	
Postgraduate degree	1.717	0.415	11.785	
No answer	0.000	0.000	Inf	
Baseline past-year substance use	8.251	1.802	25.668	0.010
Year 1 past-year substance use	44.552	14.173	125.943	<0.001
Ethnicity × baseline BIS scores	1.195	1.038	1.378	0.013

## Discussion

Elevated levels of BI may contribute risk for both anxiety disorders and substance use disorders. BI has been shown to concurrently and prospectively predict anxiety, while the association between BI and substance use has been mixed. It is possible that the relationship between BI and substance use varies by social and contextual factors. H/L youth in particular may have stronger relationships between BI, anxiety, and substance use. The present study evaluated (1) the prospective relationships between BIS scores, anxiety, and substance use in youth across 1- and 2- year follow-ups of the ABCD study and (2) whether these relationships differed by H/L ethnicity. Results indicated that baseline (ages 9–10) levels of BIS scores prospectively and positively predicted anxiety symptoms at both 1- and 2-year follow-ups (ages 10–12). The relationship between baseline levels of BIS and follow-up levels of anxiety did not differ by ethnicity. Baseline (ages 9–10) levels of BIS also prospectively predicted increased likelihood of substance use at 2-year follow-up (ages 11–12), but only for H/L youth and not for non-H/L youth. No main effects of, or interactions between, ethnicity and BIS scores were found on substance use at the 1-year follow-up.

The results showing that baseline BIS scores prospectively and positively predicted anxiety symptoms across the follow-ups are consistent with prior literature on the relationship between BI and anxiety (17, 23–28). While prior studies have shown that H/L youth report higher levels of anxiety than non-H/L youth (45–51), the present study did not find any ethnic differences in the strength of the relationship between BI and anxiety. It is possible that ethnic differences in anxiety depend on the measure of anxiety (57). H/L are more likely to experience and report physiological symptoms of anxiety (48, 70). The CBCL DSM-5 anxiety problems scales may not best represent H/L youth’s experience of anxiety. Additionally, other risk factors for anxiety may play a more important role in H/L youth’s experience of anxiety and better explain the ethnic differences in anxiety in youth. For example, individual differences in sensitivity to uncertain threat may be a stronger predictor of anxiety, and particularly for H/L (71–74).

Results related to the relationship between BIS scores and substance use varied across the follow-up years. The lack of association between BIS scores and likelihood of substance use at 1-year follow-up may be due to the fact that substance use at 1-year follow-up was infrequent and did not greatly increase from baseline. While overall substance use increased at 2-year follow-up in the sample, BIS scores only predicted increased likelihood of substance us in H/L youth (as compared to non-H/L youth). Similar to the results of Morris et al. (40), these results were independent of levels of BAS scores. This finding is also consistent with results from Chen and Jacobson (44) showing that H/L youth have the highest initial rates of substance use. H/L youth’s increased exposure to crime, community violence, chronic stress, and racial discrimination (52–56) may also increase coping and conformity motives which in turn may increase likelihood of substances use. It is possible that high BI, in addition to, or in conjunction with, additional risk factors such as increased access to substances, reduced parental monitoring, and association with deviant peers (75) may uniquely contribute to risk for early use of substances in H/L. Further research is needed to understand whether and how such risk may change as rates of substance use change across development.

The present study had several limitations and future directions. While the longitudinal nature of the ABCD study allowed for the investigation of prospective and not just concurrent relationships between BIS scores, anxiety, and substance use, it is possible that the age of the sample at baseline and through the follow-ups is still too early to best capture these relationships. As BI is often first assessed in infancy or early childhood (18, 22), the strength of the relationships between BI, substance use, and anxiety may vary across development and the lifespan. Relatedly, assessing BI via behavioral observation in infancy or early childhood may yield different results than the self-reported BIS scale scores utilized in the present investigation. Additionally, as rates of substance use increase across adolescence and early adulthood and use trajectories vary between ethnicities (44), the relationships between BIS scores, ethnicity, and substance use may vary based on the time point in which substance use is measured. These relationships may also vary across H/L youth and could differ based on factors such as time living in the US, social stigma, acculturation, language, nativity, and socioeconomic status (48, 76–83). Lastly, the ABCD study sample is not a clinical or treatment seeking sample and utilizing clinical samples may impact the strength of the relationships explored in the present study. Additional prospective studies are needed to understand how BIS scores and ethnicity relate to substance use as use increases in future follow-up years of the ABCD Study. Additional research is also needed to understand how factors such as trauma exposure, stress, cultural values, discrimination, coping motives, conformity motives, etc. may mediate the relationship between BI and substance use in H/L youth.

In conclusion, high levels of BIS prospectively predict increased rates of anxiety symptoms in both H/L and non-H/L youth. However, BIS scores uniquely predict increased likelihood of substance use for H/L youth. Future studies are needed to further understand the mechanisms ‘underlying the relationship between BI and substance use in H/L youth that will provide a scientific basis to better inform prevention and intervention programs for the H/L community.

## Data availability statement

The datasets presented in this study can be found in online repositories. The names of the repository/repositories and accession number(s) can be found below: https://nda.nih.gov/abcd/.

## Ethics statement

The studies involving humans were approved by the University of California, San Diego Institutional Review Board. The studies were conducted in accordance with the local legislation and institutional requirements. Written informed consent for participation in this study was provided by the participants’ legal guardians/next of kin.

## Author contributions

ED, AW, and WP substantially contributed to data acquisition, analysis, and interpretation of data, provided critical revisions, and agreed to be accountable for all aspects of the work in ensuring that questions related to the accuracy or integrity of any part of the work are appropriately investigated and resolved. JJ provided substantial contributions to the conception and design of the work, the acquisition, analysis, interpretation of data for the work, as well as critical revisions for important intellectual content, and agreed to be accountable for all aspects of the work in ensuring that questions related to the accuracy or integrity of any part of the work are appropriately investigated and resolved. All authors contributed to the article and approved the submitted version.
